# Investigation of the performance of a horizontal-axis dual rotor wind turbine

**DOI:** 10.1038/s41598-024-55844-6

**Published:** 2024-03-14

**Authors:** Dina Ahmed Hosni Salah, Mahmoud Abed El-Rasheed Nosier, Ashraf Mostafa Hamed

**Affiliations:** Faculty of Engineering, Ain Shams University, Cairo, Egypt

**Keywords:** Wind, Dual rotor wind turbine, Tip speed ratio, Phase shift angle, Single rotor wind turbine, Eppler, Electrical and electronic engineering, Energy infrastructure, Mechanical engineering

## Abstract

Recent years have seen a rise in interest in wind energy as a useful alternative to harmful energies like fossil fuels. The dual rotor wind turbine (DRWT) offers more rapid rates of wind energy extraction. The current study intends to compare the performance of the turbine with and without the addition of a second rotor. Additionally, it examines how tip speed ratio and phase shift angle will affect DRWT performance. Realizable k-shear stress transport turbulence models are used to solve the three-dimensional, turbulent, stable, and incompressible flow equations for the performance of dual-rotor wind turbines. Domain-independence tests and an impartial mesh test are run to assess the results and ensure their accuracy. The researcher relies on previous studies while constructing the single rotor wind turbine model. This model uses an S826 airfoil. The front and rear rotors are given streamlined representations using ANSYS, according to the researcher. The independent mesh test indicates that the mesh density has 11.5 million elements. The experiment's results show that the DRWT has a significant effect on the efficiency of wind energy.

## Introduction

Since wind energy has the potential to be an unending source of energy, its significance in the world's energy production has increased^1^. The intelligent utilization of wind power can lead to sustainable replacements for fossil fuels and reduced carbon dioxide emissions^2^. Even though wind energy only makes up around 4% of the nation's electricity, installed wind generating capacity is growing significantly. According to the U.S. Department of Energy (DOE), wind energy might supply 20% of all electricity in the country by 2030^3^.

A single-rotor wind turbine (SRWT), the most prevalent type of wind turbine, has a rotor with three blades and a hub at the top of the tower^4^. Researchers are enhancing the efficiency of single-rotor wind turbines by enhancing blade design and enlarging rotor and tower diameters to achieve higher wind speeds^5^. Large rotors require significant area and strong wind current for power generation, posing issues like blade surface pressure, vibration load, and loading noise due to aerodynamic and gravity stresses^6,7^. Unfortunately, due to poor aerodynamics, even the most modern SRWT devices can only capture (about) 50% of the wind's potential energy^8,9^. Researchers developed and refined the idea of DRWTs to provide inexpensive energy and maximize wind turbine power production from a given surface area^10,11^. The use of two rotors aligned in a row and placed back-to-back improves the ability of DRWTs to collect energy from a certain swept region^12,13^. The DRWT system employs counter-rotating technology, allowing the downwind rotor to benefit from the swirl generated by the upwind rotor's interrupted flow^14,15^. As a result, the downwind rotor may be able to capture the additional kinetic energy associated with the swirl element in the wake stream^3,16^.

However, the DRWT is barely even close to having twice the Cp of the SRWT. Due to the interaction between the two rotors, the second rotor has the effect of reducing the front rotor's performance. Yet, the rear rotor's Cp decreased as a result of the front rotor's axial velocity decay. Turbulence also causes a sharp decline in the rear rotor's performance^17^.

Although the popularity of multiple-rotor wind turbines (MRWT) is rising quickly, the design's expanding rotor size poses many difficulties^18,19^. MRWTs' effectiveness depends on the number of small turbines and the tower's weights and deflections, which affect the rotor's inherent frequencies^20^. The torsional strength of the tower is essential since the two rotors do not rotate simultaneously^21^. The optimal spacing for wind turbines is crucial for optimal performance, but they may not be suitable for urban environments due to their size, weak winds, and air interference^22^. Wind power faces challenges due to potential unpredictability, and meteorologists forecast strength by analyzing wind speed, energy, and ambient temperature correlations^23^. To manage wind turbine efficiency, monitoring and regulating factors like the tip-speed ratio are crucial, as each blade has a distinct speed-to-tip ratio^24^. High rotor tip velocity can lead to increased blade turbulence, affecting the blade's subsequent impact^25^. The performance of a wind turbine is evaluated using critical characteristics like torque and power coefficient^26,27^. At a specific tip speed ratio, monitoring the torque coefficient enables one to determine the wind turbine's power coefficient^28^.

For this instance, Rahmatian et al.^29^ looked into how the distance between the rotors and their diameters affected DRWT. The findings demonstrate that when an auxiliary rotor of the same diameter is added to a duct, the rotary part's power coefficient rises to 16%. Furthermore, compared to two rotors with the same diameter, the power coefficient will rise by up to 13% if the two rotors have different sizes (the front rotor has a smaller diameter). Also, Taghinezhad et al.^30^ examined and evaluated the performance of dual-rotor wind turbines placed inside a specified duct. The outcomes demonstrated that the highest power ratio for dual-rotor wind turbines under ideal conditions was around 55%. Roots et al.^31^ show that doubling a wind turbine's number of rotors enhances output. Lab testing and computer stimulation were used to determine that there was an 18% increase in power. Mokhtar et al.^32^ described that the overall power coefficient shared with the rear rotor decreases as the diameter of the rear rotor increases. Additionally, as DR is raised, the efficiency of the double rotor setup declines. As the diameter of the rear rotor increases, the total area capturing wind energy decreases, while the total power dissipated coefficient decreases. With a diameter ratio of 0.75, DRWT generates 13.3% more Cp than SRWT. Moreover, the DRWT's overall performance is largely stable because the power coefficient of the front rotor rises with distance while the power coefficient of the rear rotor falls.

Bani-Hani et al.^33^ examined the performance of single-rotor, double-concurrent, and double-countercurrent, three-bladed wind turbines. The results revealed that the addition of a second rotor increased the wind turbine's efficiency. Additionally, the counter-rotating dual rotor model outperformed the single and concurrent double-rotor models in terms of efficiency. Additionally, Abdelkarim^34^ investigates the aerodynamic performance of wind turbines, aiming to maximize the power extracted from the wind. The study focuses on the effect of introducing a second rotor to the main rotor of the wind turbine in what is called a dual-rotor wind turbine (DRWT). The numerical study took place on the performance of a small-scale model of a wind turbine of 0.9 m diameter using an S826 airfoil. This research focused on the angle attack and distance effects on the turbine's performance. The results showed that the co-rotating wind turbine (CWT) and counter-rotating wind turbine (CRWT) had better performance compared to that of the SRWT, with an increase of 12–14% in peak power coefficient. Moreover, the effect of changing the pitch angle of the rear rotor on the overall performance found a negligible effect between angles 0° and 2° degrees tilting towards the front rotor.

Based on the aforementioned literature, this paper aims to analyze the efficiency of the turbine both before and after the addition of a second rotor and to comprehend how changing the rear rotor's pitch impacts the turbine's performance. This paper is considered significant as it investigates the impact of the tip speed ratio (TSR) of the two rotors on the performance of CWT. Also, it highlights the great impact of phase shift angle on the turbine power coefficient.

## Methodology

The single and dual-rotor wind turbines were designed according to Tables 1 and 2, their performance is modeled and tested using the mathematical models for CFD that are related to fluid dynamics, The entire computational framework is made through ANSYS 19.2 software, using a laptop with a processor of (11th Gen Intel(r) core(tm) i7-11800 h @2.3 GHz) and 16 GB RAM.

**Table 1:** BEM calculations for the airfoil.

S	${r}_{i}$ (m)	${\lambda }_{r,i}$	$U_{blade}\, \left( {\frac{{{\text{m}}^{2} }}{{\text{s}}}} \right)$	${W}_{rel}\,\left({\frac{{{\text{m}}}}{{\text{s}}}}\right)$	${\varnothing }_{i}\,({\text{deg}})$	${\propto }_{design}\,({\text{deg}})$	${\beta }_{i}$	Chord ${C}_{i}\,\left({\text{m}}\right)$
1	0.011	0.038	0.283	7.555	58.568	6	52.568	0.035
2	0.034	0.113	0.647	7.578	55.721	6	49.721	0.095
3	0.056	0.188	1.078	7.627	52.92	6	46.92	0.144
4	0.079	0.263	1.509	7.699	50.194	6	44.194	0.183
5	0.101	0.338	1.941	7.795	47.567	6	41.567	0.212
6	0.124	0.413	2.372	7.914	45.056	6	39.056	0.234
7	0.146	0.488	2.803	8.054	42.674	6	36.674	0.25
8	0.169	0.563	3.234	8.214	40.428	6	34.428	0.26
9	0.191	0.638	3.666	8.393	38.322	6	32.322	0.266
10	0.214	0.713	4.097	8.59	36.353	6	30.353	0.268
11	0.236	0.788	4.528	8.804	34.52	6	28.52	0.268
12	0.259	0.863	4.959	9.033	32.815	6	26.815	0.266
13	0.281	0.938	5.391	9.277	31.232	6	25.232	0.263
14	0.304	1.013	5.822	9.534	29.763	6	23.763	0.258
15	0.326	1.088	6.253	9.803	28.4	6	22.4	0.253
16	0.349	1.163	6.684	10.084	27.135	6	21.135	0.247
17	0.371	1.238	7.116	10.375	25.961	6	19.961	0.241
18	0.394	1.313	7.547	10.675	24.869	6	18.869	0.235
19	0.416	1.388	7.978	10.984	23.854	6	17.854	0.229
20	0.439	1.463	8.41	11.301	22.909	6	16.909	0.223
21	0.461	1.538	8.841	11.626	22.027	6	16.027	0.217
22	0.484	1.613	9.272	11.957	21.204	6	15.204	0.211
23	0.506	1.688	9.703	12.295	20.434	6	14.434	0.205
24	0.529	1.763	10.135	12.638	19.713	6	13.713	0.2
25	0.551	1.838	10.566	12.986	19.037	6	13.037	0.194
26	0.574	1.913	10.997	13.339	18.403	6	12.403	0.189
27	0.596	1.988	11.428	13.697	17.806	6	11.806	0.184
28	0.619	2.063	11.86	14.059	17.244	6	11.244	0.179
29	0.641	2.138	12.291	14.425	16.715	6	10.715	0.175
30	0.664	2.213	12.722	14.794	16.215	6	10.215	0.17
31	0.686	2.288	13.153	15.166	15.742	6	9.742	0.166
32	0.709	2.363	13.585	15.542	15.295	6	9.295	0.162
33	0.731	2.438	14.016	15.92	14.871	6	8.871	0.158
34	0.754	2.513	14.447	16.301	14.469	6	8.469	0.154
35	0.776	2.588	14.878	16.684	14.087	6	8.087	0.15
36	0.799	2.663	15.31	17.07	13.724	6	7.724	0.147
37	0.821	2.738	15.741	17.458	13.378	6	7.378	0.144
38	0.844	2.813	16.172	17.848	13.049	6	7.049	0.14
39	0.866	2.888	16.603	18.239	12.735	6	6.735	0.137
40	0.889	2.963	17.035	18.633	12.435	6	6.435	0.134

**Table 2:** BEM calculations for the airfoil Eppler design (E63).

S	${r}_{i}$(m)	${\lambda }_{r,i}$	${U}_{blade}\,\left({\frac{{{\text{m}}^{2} }}{{\text{s}}}}\right)$	${W}_{rel}\,\left({\frac{{{\text{m}}}}{{\text{s}}}}\right)$	${\varnothing }_{i}\,({\text{deg}})$	${\propto }_{design}\,({\text{deg}})$	${\beta }_{i}$	Chord ${C}_{i}\,\left({\text{m}}\right)$
1	0.02	0.125	0.839	7.586	55.25	5	50.25	0.062
2	0.06	0.375	2.516	7.949	46.296	5	41.296	0.133
3	0.1	0.625	4.194	8.628	38.663	5	33.663	0.158
4	0.14	0.875	5.872	9.556	32.543	5	27.543	0.158
5	0.18	1.125	7.549	10.67	27.756	5	22.756	0.149
6	0.22	1.375	9.227	11.916	24.018	5	19.018	0.137
7	0.26	1.625	10.904	13.257	21.072	5	16.072	0.125
8	0.3	1.875	12.582	14.668	18.715	5	13.715	0.114
9	0.34	2.125	14.26	16.13	16.801	5	11.801	0.104
10	0.38	2.375	15.937	17.631	15.222	5	10.222	0.096
11	0.42	2.625	17.615	19.161	13.903	5	8.903	0.089
12	0.46	2.875	19.292	20.713	12.786	5	7.786	0.082
13	0.5	3.125	20.97	22.284	11.83	5	6.83	0.076
14	0.54	3.375	22.648	23.87	11.003	5	6.003	0.071
15	0.58	3.625	24.325	25.467	10.281	5	5.281	0.067
16	0.62	3.875	26.003	27.074	9.647	5	4.647	0.063
17	0.66	4.125	27.68	28.689	9.085	5	4.085	0.06
18	0.7	4.375	29.358	30.311	8.583	5	3.583	0.056
19	0.74	4.625	31.036	31.938	8.134	5	3.134	0.054
20	0.78	4.875	32.713	33.571	7.728	5	2.728	0.051

### SRWT model

**Figure 1:**
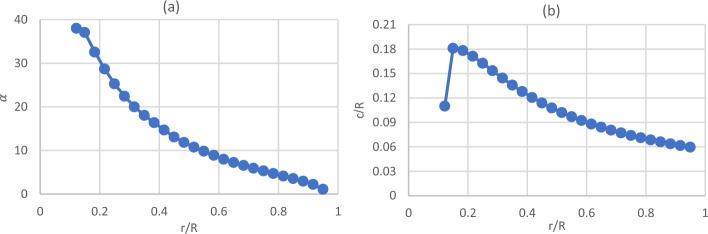
Blade definition by (**a**) twist angle as a function of the radius and (**b**) chord length as a function of the radius.

**Figure 2:**
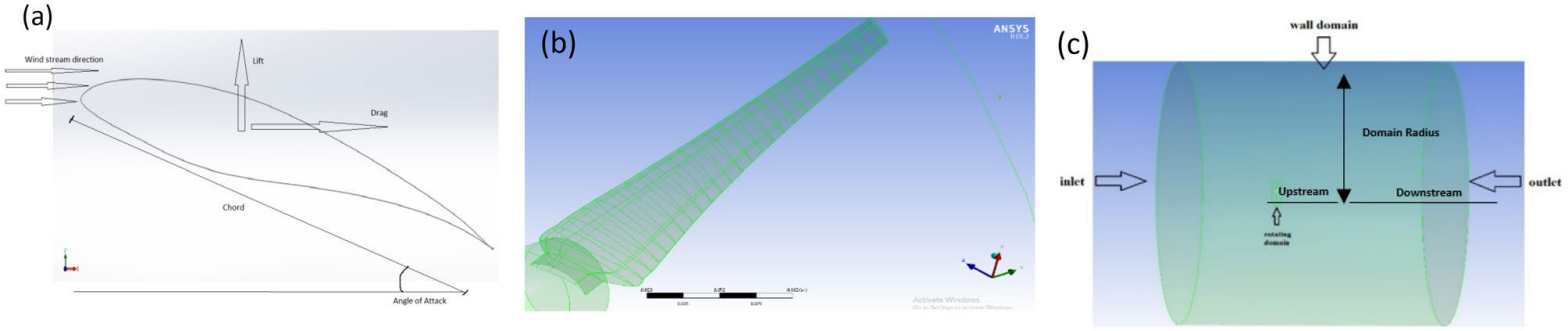
SRWT model (**a**) airfoil (s826) used in blade's sections by Krogstad and Lund ^34^, (**b**) airfoil model constructed by ansys, and (**c**) rotating domain.

The construction of the SRWT turbine model is believed to be the initial step in the numerical analysis. The experimental work on 0.9 m SRWT conducted by Krogstad and Lund^35^ was used to review the SRWT. By employing a newly developed BEM approach and correcting tip losses with Prandtl and the force with Glauert, the authors were able to specify the blade chord and twist across various radii, as shown in Fig. 1a and b. Figure 2a depicts the S826 airfoil that was employed across the blade radii. Figure 2b also shows the airfoil model made by ANSYS.

Following numerical research utilizing BEM theory and CFD, the simulation's effectiveness was evaluated at an upwind speed of 10 m/s. It was reported that the CFD model is the best for figuring out the drag and lift coefficients (Cd and Cl) of wind turbines because it was developed utilizing the MRF technique and the K-SST turbulence model. Additionally, the ANSYS simulation model's airfoil was separated into ten splines to improve the Cp value and the airfoil model in the leading mesh type. Two domains make up the MRF approach: a stationary domain that takes up a third of the main domain and a 180° domain. The performance coefficient is measured for some configurations that have different upstream, downstream, and domain radii. Figure 2c depicts these configurations and the domain arrangement with the symmetrical boundary condition covering the inlet and exit sides. According to the domain size that provides the best performance with unaffected Cp, the upstream radius should be 4.05 R, the downstream radius should be 15, and the domain radius should be 6 R as a result of the main domain independence test (see Table 3). Moreover, the domain spins at the same speed as the blade. Testing that is independent of either domain is done on both.

**Table 3:** The primary domain independence test's primary domain sizes.

Configurations	Upstream (R)	Downstream (R)	Domain radius (R)	Cp
1	3.5	9.5	5	0.412
2	3.5	9.5	6	0.384
3	4.05	12	6	0.365
4	4.05	15	6	0.364
5	5	16	7	0.364
6	5.5	17	7	0.364

**Figure 3:**
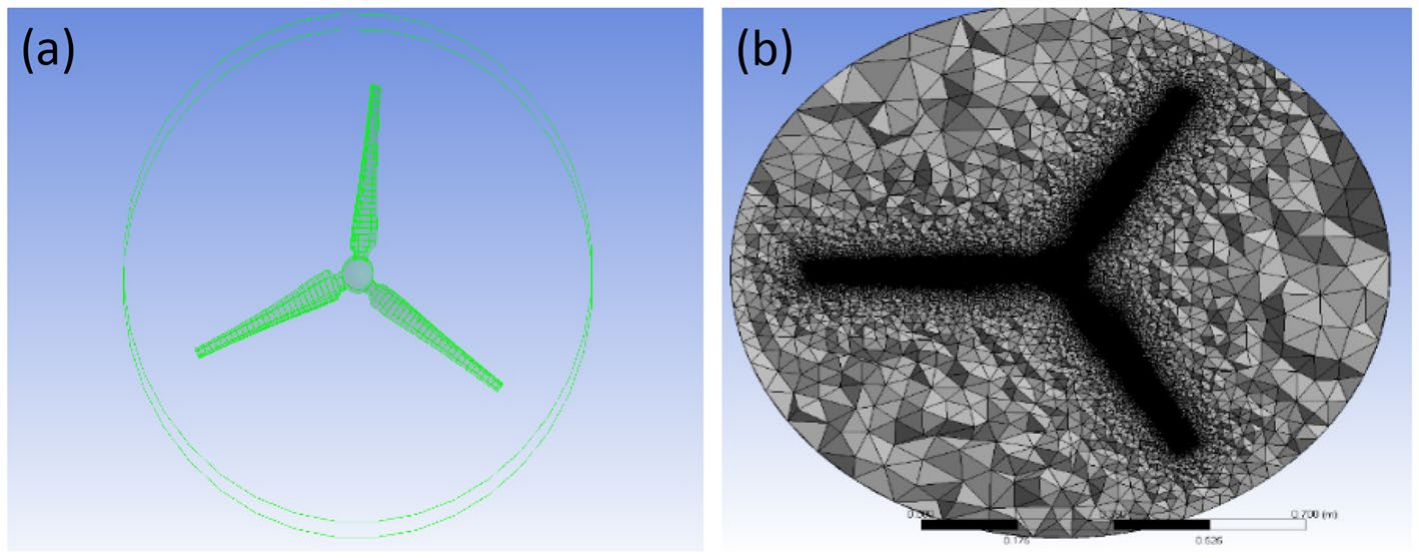
Blade definition by (**a**) twist angle as a function of the radius and (**b**) chord length as a function of the radius.

Additionally, a domain-independence test is run on the rotary domain. The findings demonstrate that, between a rotating domain extrusion of 0.17 of the rotor radii and a rotating domain radius of 0.5 of the rotor radii, the rotating domain size expansion does not affect the rotor efficiency (see Table 4). Figure 3a depicts the rotating domain configuration in ANSYS's CFD simulations of the SRWT. Mesh density has a considerable impact on the CFD simulation results. The mesh is concentrated on the periodic domain's outer boundaries as well as the influenced body around the blade. The blade boundaries must be expanded to guarantee the detection of turbulence near the blade surface and to get Y-plus as close to zero as possible.

**Table 4:** Rotating domain sizes studied in the rotating domain independence test for the SRWT.

Configurations	Upstream (R)	Downstream (R)	Domain radius (R)	Cp
1	0.13	0.13	1.33	0.285
2	0.17	0.14	0.4	0.3
3	0.17	0.17	0.5	0.32
4	0.17	0.18	0.5	0.32

**Figure 4:**
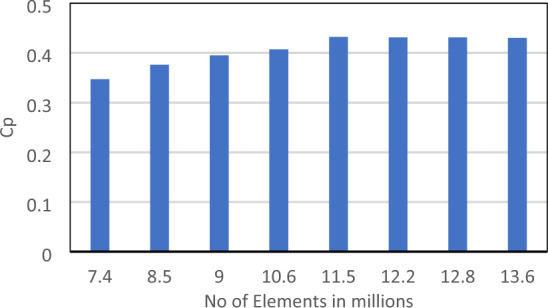
Cp versus mesh density.

The mesh independence was tested at a TSR of 6. According to the mesh independence test, the mesh density has a number of elements of 11.5 million (see Fig. 4). The Cp at TSR of 6, according to the experiment's findings conducted by Krogstad and Lund^35^, was equivalent to 0.4375, which is comparable to the outcome of this present case study employing the K-ω SST model. In this model, Cp at TSR of 6 for the present turbulence model was 0.432. Figure 3b shows a model for the turbine in the mesh, which is constructed using ANSYS. In order to acquire the necessary precise findings and enable the model to examine the turbulence occurrence close to the blade geometry, Krogstad and Lund^35^ stressed the need to keep the value of Y+ close to zero. Lower Y+ results are produced by increasing the mesh density in the vicinity of the blade. The region with more than one Y+ close to the blades was refined throughout the solution in order to make further adaptations. Utilizing the mesh control's inflation option will allow you to regulate the adaptation. By choosing the mesh initial layer position, it is possible to manage the inflating layers close to the blade to be as close as required.

### DRWT model

**Figure 5:**
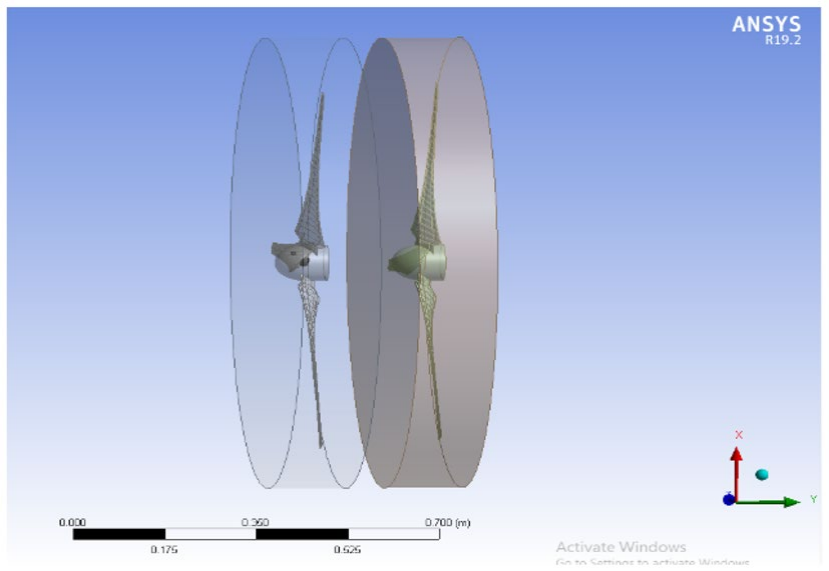
DWRT geometry with front and rear rotors by ANSYS.

**Figure 6:**
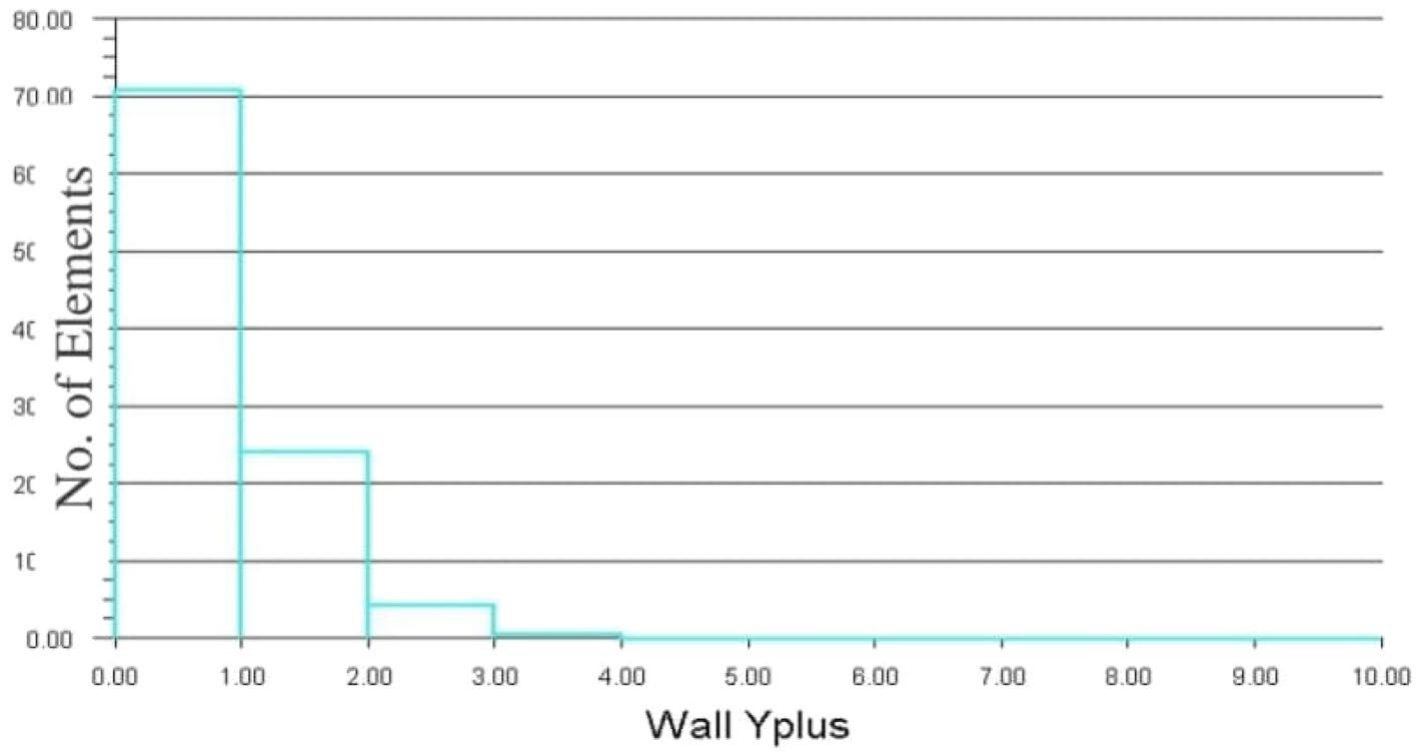
Relation between no. of elements and wall Y plus.

**Figure 7:**
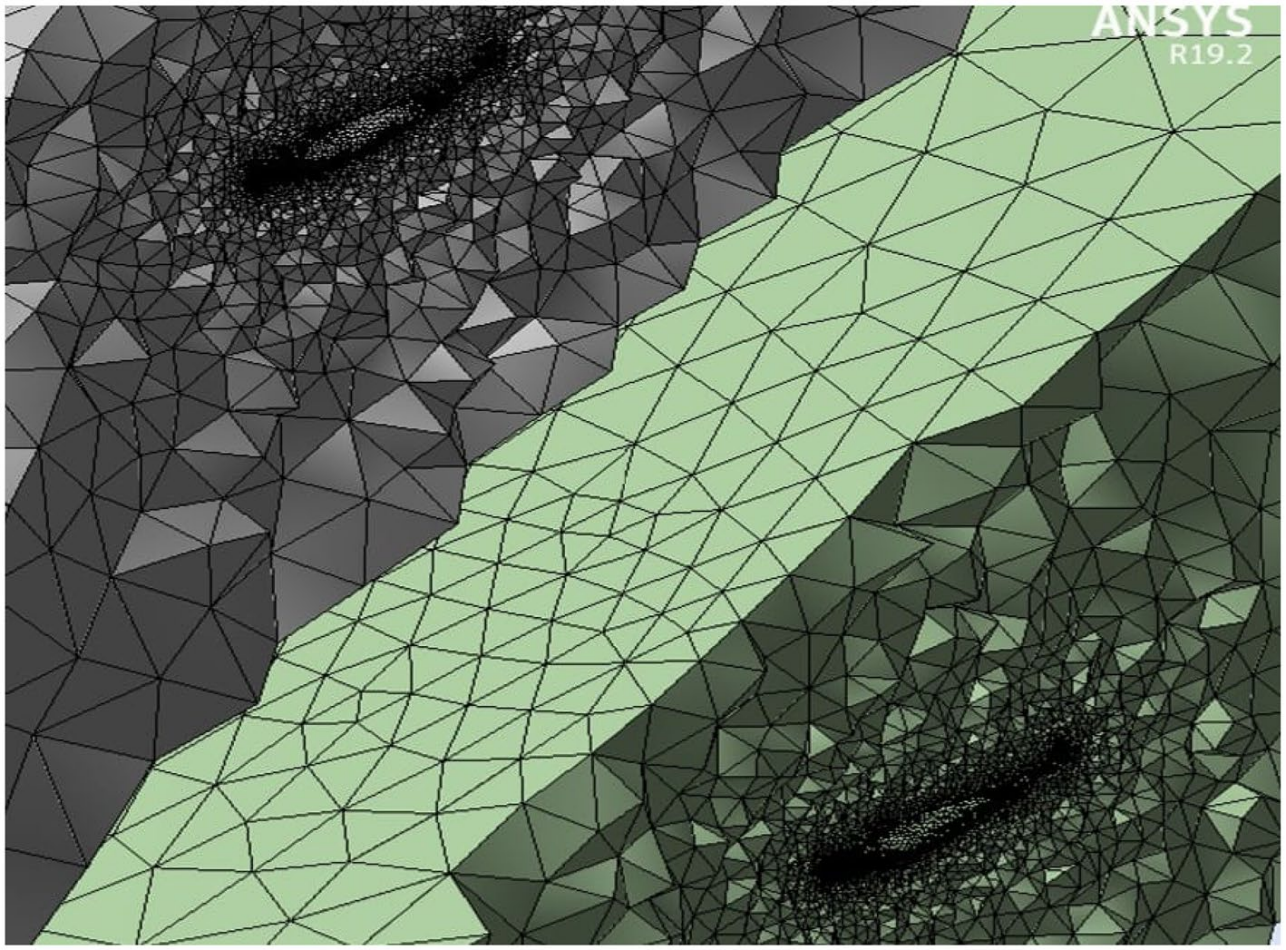
Flow pattern analysis of the CUUT.

**Figure 8:**
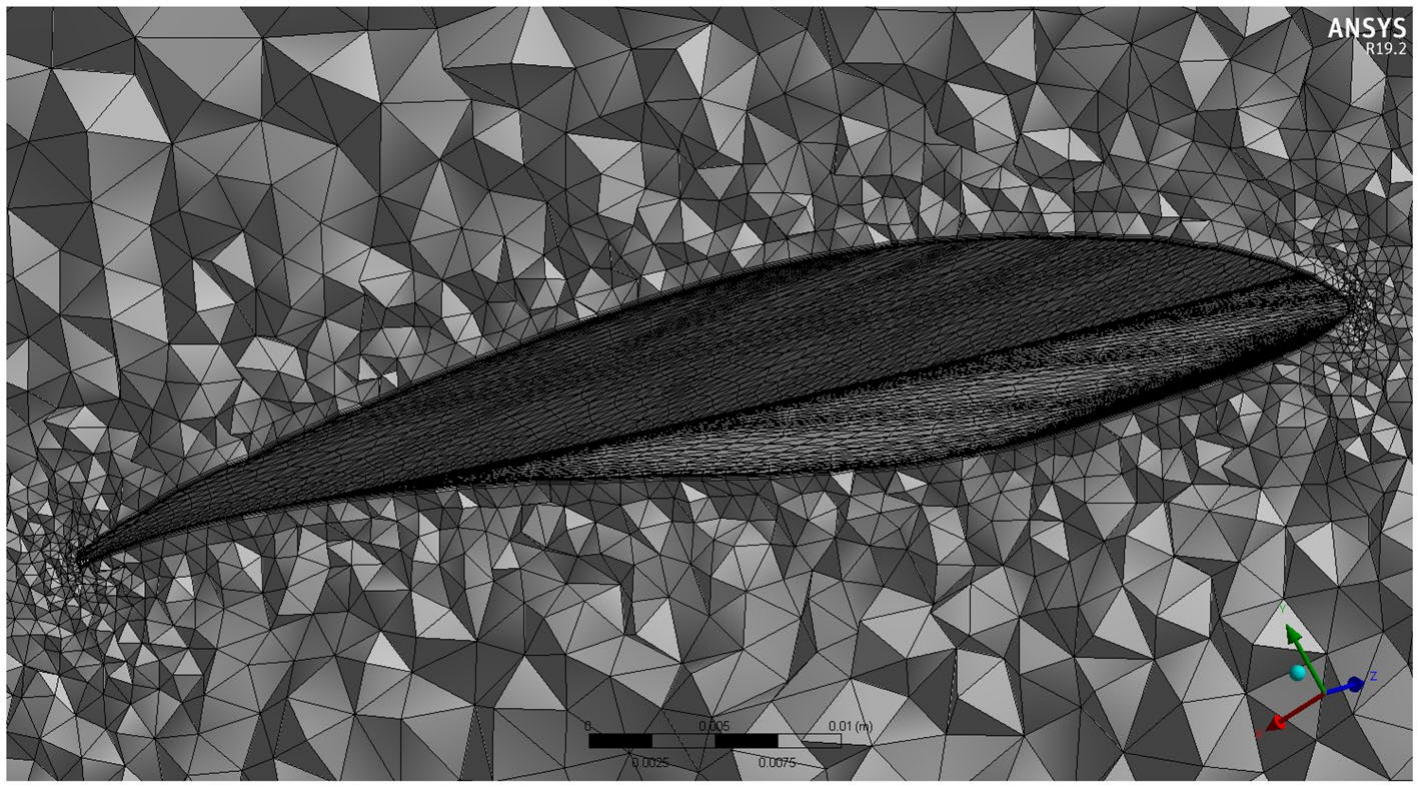
Mesh at air foil section.

**Figure 9:**
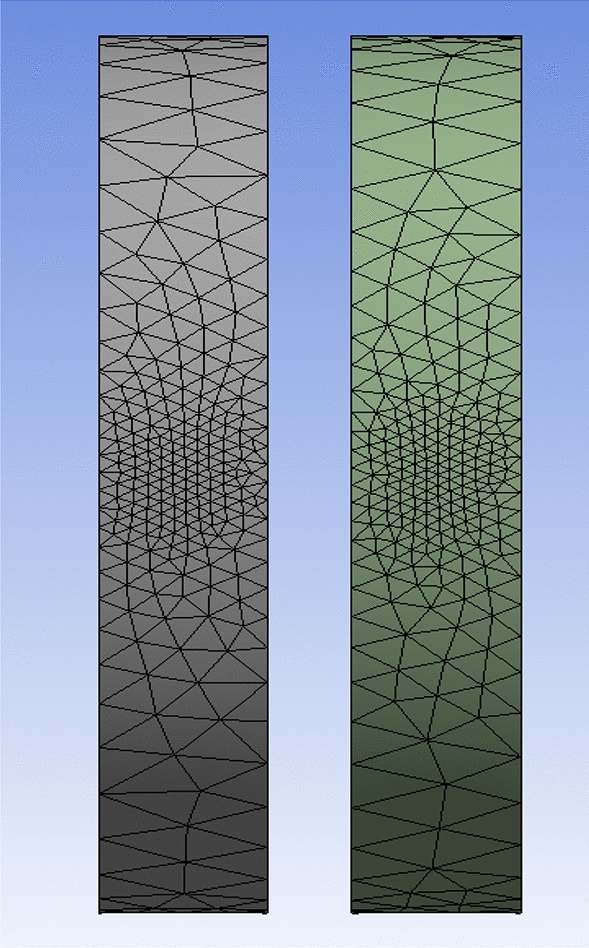
Inner domain mesh.

**Figure 10:**
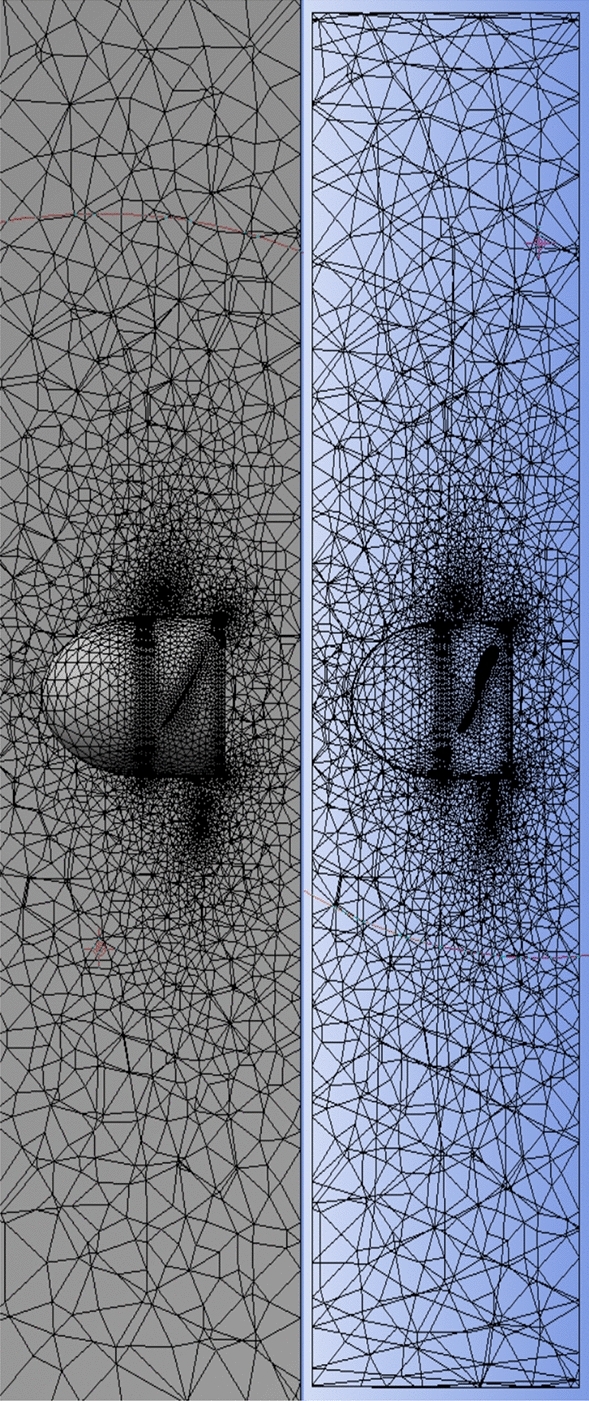
Mesh view at half of domain.

**Figure 11:**
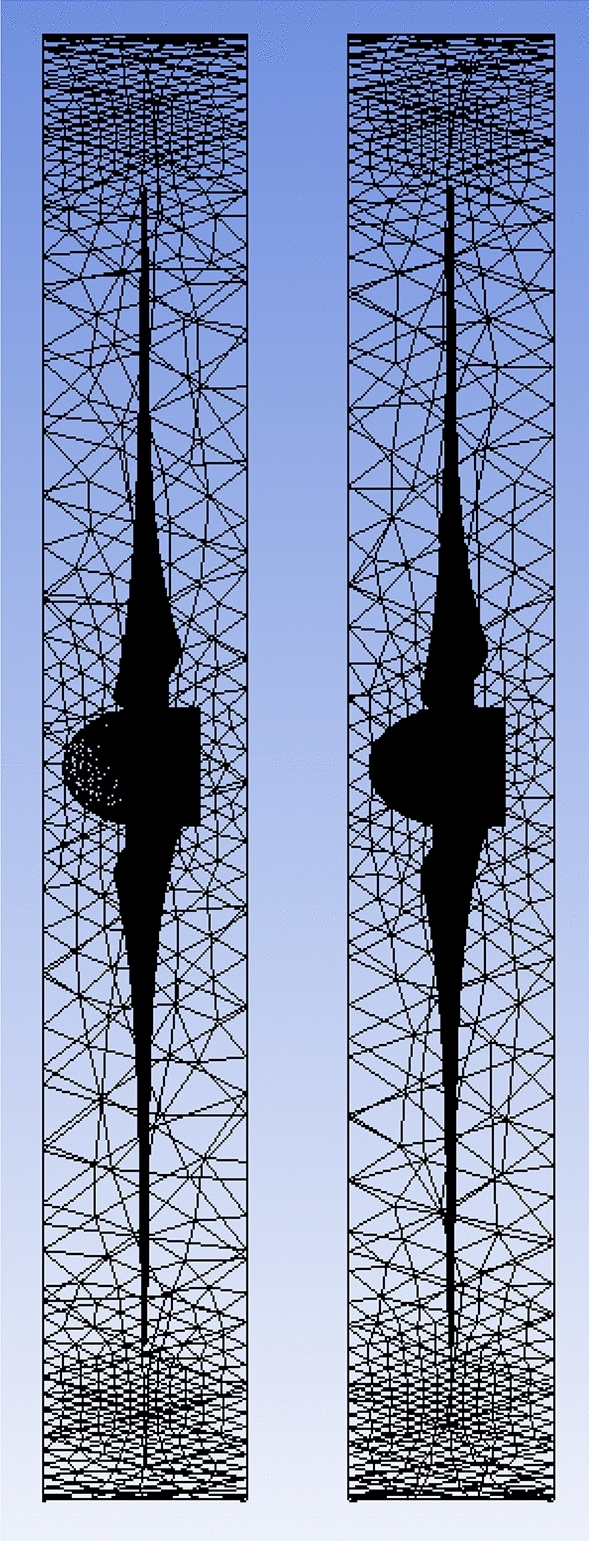
26Inner domain mesh CWT.

**Figure 12:**
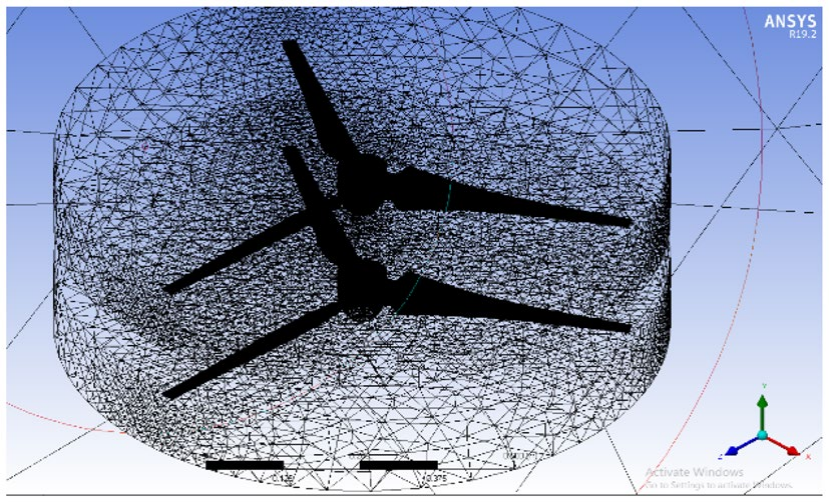
A mesh model for CWT using ANSYS.

The front and back rotors of the DRWT model are positioned in a row, have the same diameter, and rotate at the same speed, with a gap of 0.5 R between them. The diameter of each rotor is 0.9 m. As shown in Fig. 5, the domain with two rotors has a diameter ratio of 1. The input rotational speed will differ for each of the two rotating domains in the main domain as well as the body of influence surrounding the turbine, where the mesh density is higher. The domain size and mesh will be the same for the DRWT analysis with a diameter ratio of 1:1. To make sure that the primary domain won't need to be expanded to accommodate the addition of the rear rotor to the SRWT, a domain-independence test is done on it. The domain independence test is run at TSR 6, when Cp starts to become unaffected by the primary domain size expansion,the length of the domain upstream of the rotor corresponds to 4.5 times the radius of the rotor, whereas the length downstream the rotor corresponds to 16 times the radius, There were five layers with 0.00017 inflation, the growth rate was 1.2 as a default, the maximum Y+ was 70 as shown in Fig. 6, The dual-rotor case's flow pattern was examined. And as shown in Fig. 7 everything was fine, we also refine the wake of the rotors as shown in Figs. 8, 9, 10 and 11. The mesh independence test is also carried out. The domain size determined by the domain independence test is used to execute the mesh independence test at TSR 6. According to the mesh independence test, the mesh density has several elements of 23.4 million. For the current turbulence model in this model, Cp at a TSR of 6 was 0.425. Figure 12 depicts a model for the CWT in the mesh, which is constructed using ANSYS.

After conducting the domain and mesh tests and investigating the DRWT model, the study will overview and discuss the effect of TSR and phase shift angle on the performance of DRWT as follows:

- Model validation for SRWT model.
- Effect of TSR and phase shift angle for rear rotor on CWT performance.
- Effect of spacing between the rotors on DRWT performance.
- Effect of diameter ratio between the front and rear rotor on DRWT performance.
- Effect of the airfoil type of rear rotor on DRWT performance.

## Results and discussion

This section concerns showing the results of the model validation of the single rotor wind turbine (SRWT) and investigating the dual rotor wind turbine (DRWT) model results.

### Model validation for SRWT model

**Figure 13:**
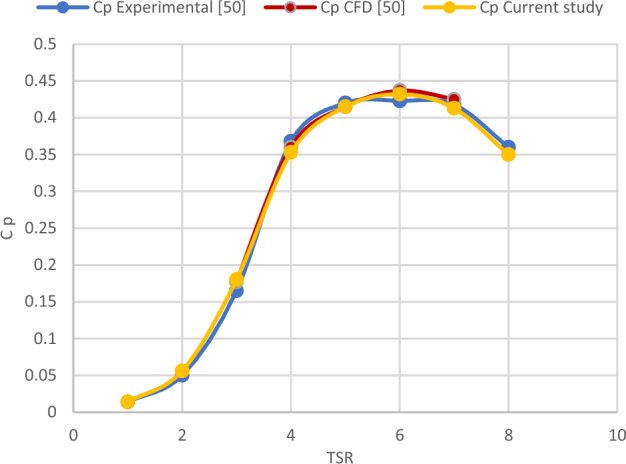
Comparison of Cp versus TSR between previous work done experimentally and with CFD vs present study^34^.

**Figure 14:**
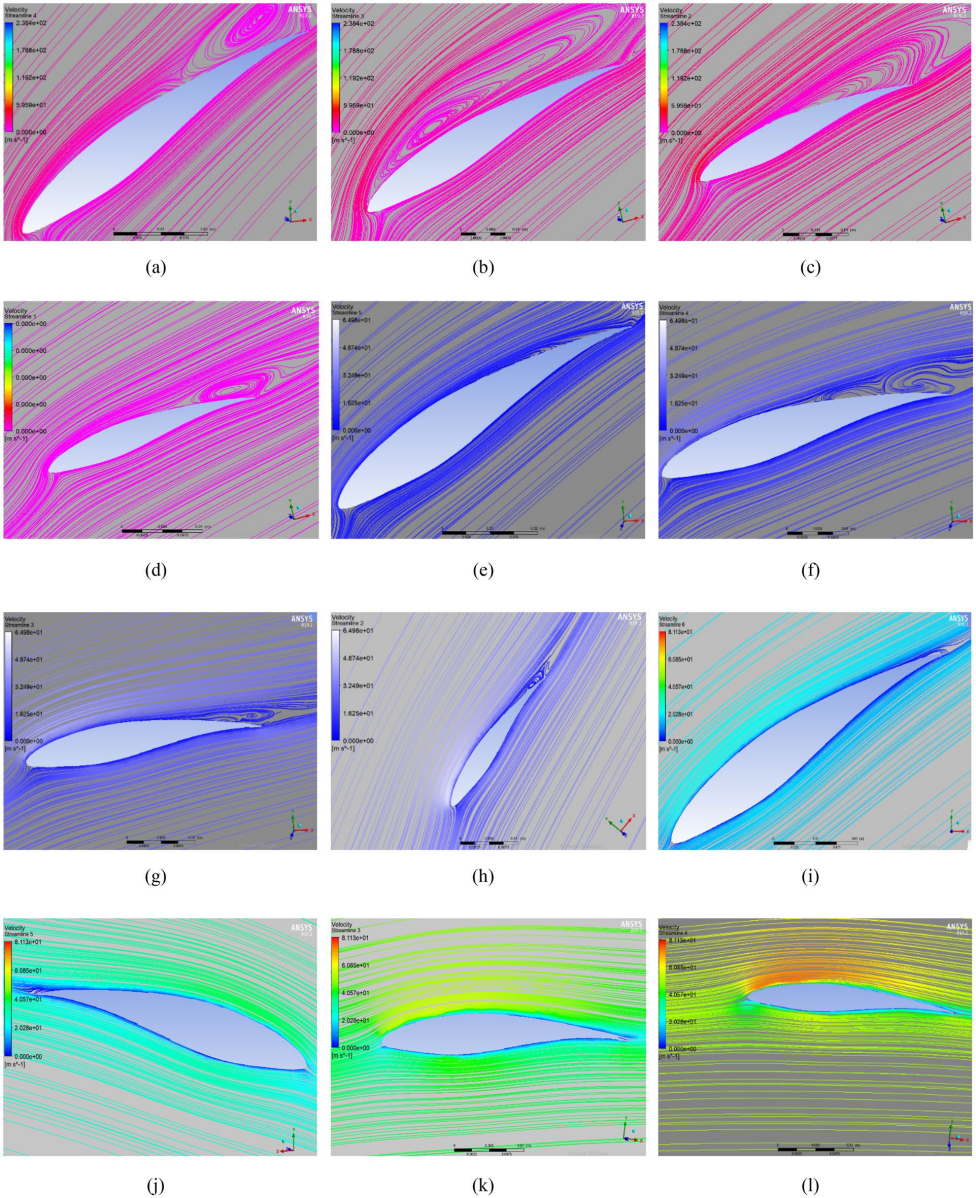
Streamlines representation for SRWT at: (**a**) TSR 3 and 0.1R (**b**) TSR 3 and 0.2R (**c**) TSR 3 and 0.3R (**d**) TSR 3 and 0.4R (**e**) TSR 4 and 0.1R (**f**) TSR 4 and 0.2R (**g**) TSR 4 and 0.3R (**h**) TSR 4 and 0.4R (**i**) TSR 6 and 0.1R (**j**) TSR 6 and 0.2R (**k**) TSR 6 and 0.3R (**l**) TSR 6 and 0.4R.

The CFD simulation findings are validated using earlier work by Krogstad and Lund^35^ at various tip speed ratios (from 1 to 8). For SRWT from 1 to 8, CFD-covered TSR is used in the simulations. Figure 13 shows that the CFD simulation follows the same general pattern as the experimental and CFD previous work with small error as the mean absolute percentage error (MAPE) for experimental and CFD were 4.05 and 1.52% respectively. Which may be related to the change in Y plus value within the allowed range. Additionally, this previous work may not have adequately taken into account the obstruction effect in the wind tunnel. The peak Cp value at TSR of 6 is the same in both the present analysis and the earlier work, and the variance is incredibly minor. Figure 14 depicts the velocity streamlines streaming at various points in the radial orientation from a radius of 0.1 m to 0.4 m around the blade at TSR values of 3, 4, and 6. For various TSRs, it is observed that as the distance from the center increases (from 0.1R to 0.4R), the velocity of the wind increases. This ensures that the area around the center of the turbine will not generate power, and the tip contributes a larger portion of the power. For a specific TSR, it is observed that the velocity of wind around the blade at TSR of 6 is higher than its value at TSR of 3 and 4.

To sum up, these graphs demonstrate linked streamlines to the blade boundary, supporting the simulation findings' excellent coefficient of performance at a TSR of 6. In contrast, the streamlines encircling the blade surface shift in the radial location at TSR 4 indicating the creation of turbulence and vortices about the blade's poor performance at that particular TSR.

### CWT model results

This section compares how well CWT and SRWT perform at various TSRs and phase shifts.

#### Phase shift angle for rear rotor

**Figure 15:**
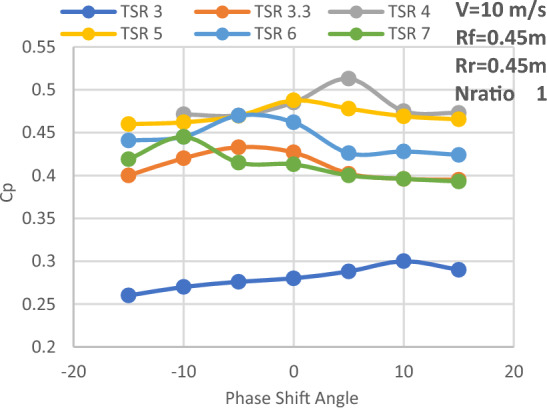
Power coefficient of CWT versus rear rotor phase shift angle at various TSRs.

The investigation of front and rear rotor performance is carried out by varying the rear rotor phase shift angle at N_ratio_ = 1. This study also uses the required velocity of 10 m/s, a diameter ratio of 1, and a variety of TSRs, including 3, 3.3, 4, 5, 6, and 7. Equations from (1) to (5) of the momentum theory^36^ are used to compute the power coefficient of the CWT vs the rear rotor phase shift angle at various TSRs. The result is displayed in Fig. 15. Additionally, Table 5 displays the phase shift angle at which each value of TSR produces the largest value of Cp.

1 $${\text{P}}_{{{\text{front}}}} = {\text{T}}_{{{\text{front}}}} * \omega$$

2 $${\text{P}}_{rear} = {\text{T}}_{rear} * \omega$$

3 $${\text{CP}}_{{{\text{front}}}} = { }\frac{{{\text{P}}_{front} }}{{\frac{1}{2}{\rho A}_{{{\text{front}}}} {\text{v}}^{3} }}$$

4 $${\text{CP}}_{rear} = { }\frac{{{\text{P}}_{rear} }}{{\frac{1}{2}{\rho A}_{{{\text{rear}}}} {\text{v}}^{3} }}$$

5 $$CP_{total} = CP_{{{\text{front}}}} + { }CP_{rear}$$

where: ${{\text{P}}}_{{\text{front}}}$: is the extracted power from the front blade, ${{\text{P}}}_{{\text{rear}}}:$ is the extracted power from the rear blade, T: Torque, $\upomega$ : rotational speed, ${\text{A}}$: The swept area of the second rotor, ${\text{V}}$: velocity wind speed.

**Table 5:** Phase shift angle which yields the maximum value of Cp at each value of TSR.

Tip speed ratio	Phase shift angle (degree)	Cp value
3	10	0.3
3.3	− 5	0.433
4	5	0.513
5	0	0.4877
6	− 5	0.47
7	− 10	0.445

#### Spacing between the rotors

**Figure 16:**
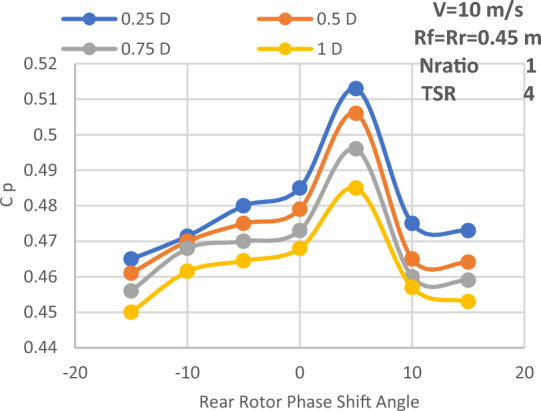
Power coefficient change with axial distance and rear rotor phase shift angle.

**Figure 17:**
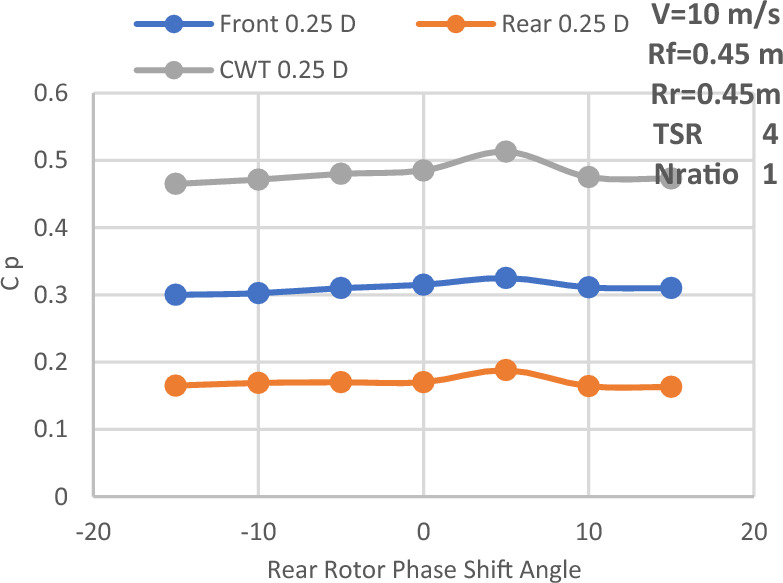
Power coefficient variation with phase shift angle at axial separation distance of 0.25D.

**Figure 18:**
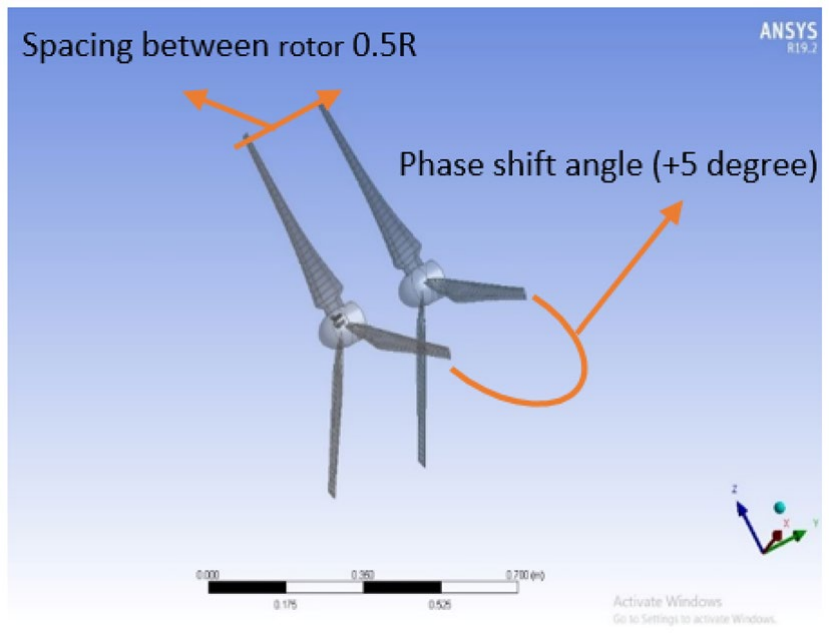
CWT configuration with 0.5R axial spacing and phase shift angle of 5° for the rear rotor using ANSYS.

The main reason for the axial distance study is that the power coefficient would be affected by the upstream rotor's swirl, which varies in both velocity and direction with axial distance. The power coefficient variation with the rear rotor phase shift angle for different axial distances between the two rotors is displayed in Fig. 16. Additionally, this study is conducted at the suggested speed of 10 m/s, with a N_ratio_ of 1 and a diameter ratio of 1, as well as an optimal TSR value of 4. Additionally, Fig. 16 makes it evident that an axial distance of 0.25D_front_ or 0.5R from the front rotor provides the highest overall performance for CWT. Thus, Fig. 17 depicts the power coefficient change at an axial distance of 0.25D_front_ for the front rotor, rear rotor, and CWT with phase shift angle. The axial distance has a negligible effect on the CWT performance, as seen by the above numbers. However, the optimal axial distance for achieving maximum performance is determined to be 0.25D overall. Furthermore, the highest Cp at the ideal axial distance of 0.25D_front_ is likewise obtained with a phase shift angle of 5°. The CWT configuration at the optimal values—0.5R or 0.25D for the axial spacing between the rotors and a 5° phase shift angle for the rear rotor—is seen in Fig. 18. ANSYS is used for the scheme of this arrangement.

#### Rotational speed ratio between the two rotors

**Figure 19:**
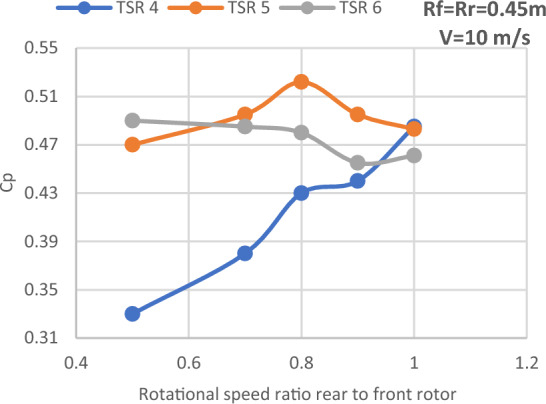
Power coefficient versus N_ratio_ for different TSR values.

Figure 19 shows the effect of the rotational speed ratio on the performance of CWT at various TSR values. Also, Table 6 depicts the maximum values of Cp at different N_ratio_ for CWT. For a TSR of 5, the rotational speed ratio usually reaches its maximum value at a speed ratio of 0.8. Finally, it was found that a rotational speed ratio of 0.5 corresponded to the peak Cp for a TSR of 6. As shown in Fig. 19, the rotational speed ratio impact at TSR 4 works better at ratios of 0.9 and 1.

**Table 6:** Maximum values of Cp at different N ratio CWT.

Tip speed ratio	Cp max	N_ratio_
4	0.485	1
5	0.522	0.8
6	0.49	0.5

### Diameter ratio between the two rotors

**Figure 20:**
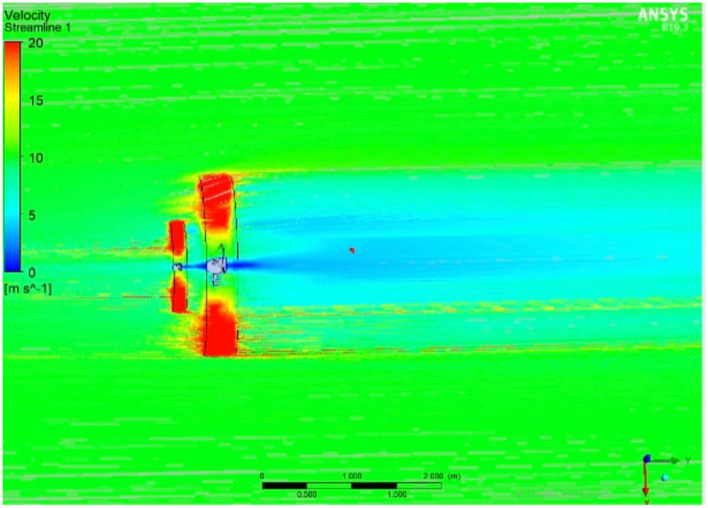
DRWT velocity analysis using ANSYS.

The influence of the diameter ratio has been investigated in several research because of the expectation that it will lead to an enhancement in the DRWT performance, that is most likely because, in addition to the energy the upwind rotor leaves behind, the downstream rotor's tip region also grabs up with some of the free streams as shown in Fig. 20. According to the earlier research in this thesis, when the front and rear rotors have a diameter ratio of one, the front rotor extracts a significantly higher percentage of the power than the rear rotor because its velocity decelerates more quickly.

#### Diameter ratio (1:2) with the same airfoil (S826)

**Figure 21:**
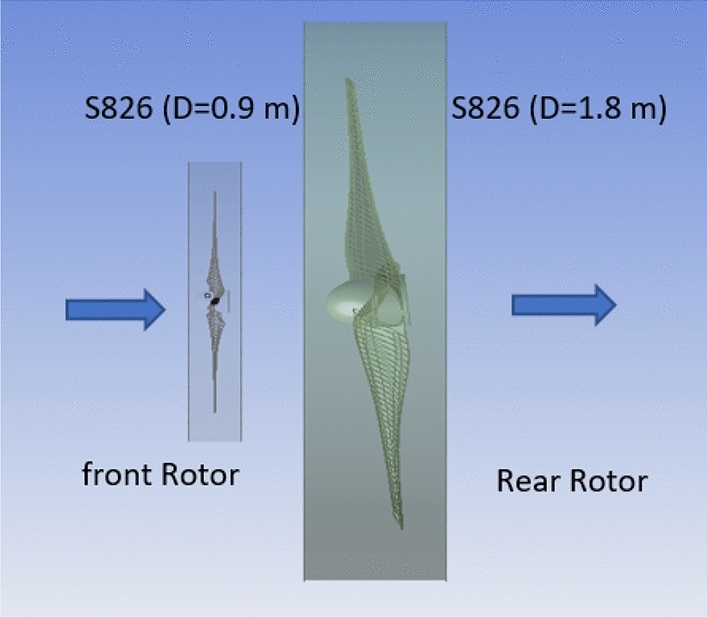
Dual rotor wind turbine with front rotor (S826, D = 0.9 m) and rear rotor (S826, D = 1.8 m).

**Figure 22:**
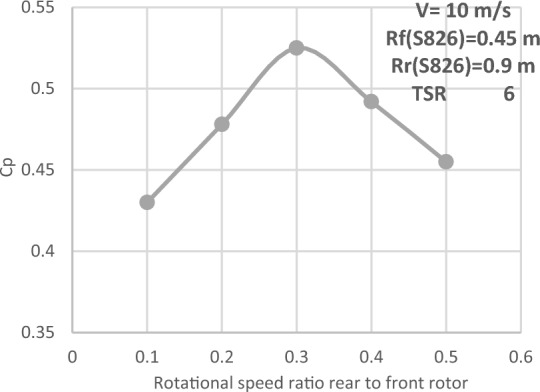
Cp versus rotation speed ratio rear (S826 D 1.8 m) to front rotor (S826 D 0.9 m) at various N_ratio_ for CWT.

A new diameter ratio was chosen to increase DRWT's efficacy. The airfoil type (S826) and diameter (0.9 m) of the front rotor remain unchanged. Using the same sort of airfoil, the rear rotor diameter is doubled to 1.8 m (see Fig. 21). At various N_ratio_ values, the impact of this diameter ratio is examined. The performance coefficient for DRWT employing a diameter ratio of 1:2 and the same type of airfoil is displayed in Table 7 and Fig. 22.

**Table7:** Performance coefficient for DRWT for diameter ratio of (1:2) with the same airfoil type (S826) at various N_ratio_ values.

TSR	N_ratio_	Cp total
6	0.5	0.455
6	0.4	0.492
6	0.3	0.525
6	0.2	0.478
6	0.1	0.43

The optimal Cp value was found to be 0.525 at TSR 6 and a N_ratio_ of 0.3, according to the data. It is noted that the Cp increased by 0.575% with increasing rear rotor diameter when compared to the best Cp achieved from the earlier experiments with a diameter ratio of (1:1), which was (0.522). As a result, using the same type of airfoil with a diameter ratio of 1:2 does not appear to improve DRWT efficiency.

#### Diameter ratio (1:2) with Eppler E63 airfoil for rear rotor

**Figure 23:**
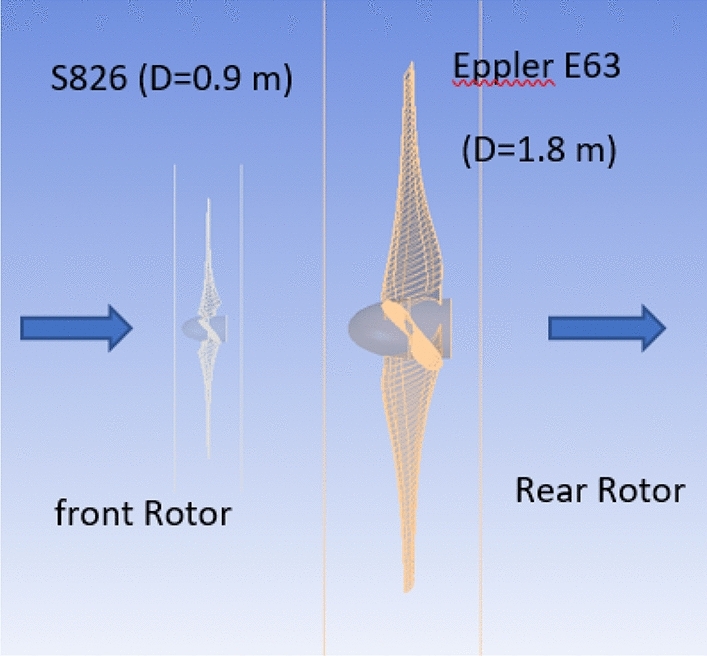
DRWT configuration with front rotor (S826 airfoil and D 0.9 m) and rear rotor (Eppler airfoil and D 1.8 m).

When the diameter ratio was changed to 1:2 using the same airfoil, the outcomes were insufficient. As a result, in an additional attempt to improve DRWT performance, the type of airfoil for the rear rotor is modified. When the Eppler type of airfoil was compared to SRWT using the S826 airfoil, the power coefficient increased by 43%. Furthermore, an Eppler airfoil with a 1.8 m diameter has previously been designed for SRWT. Thus, the Eppler airfoil was included in the DRWT model by the researcher. The proposed arrangement, which is DRWT with a front rotor (S826 airfoil and D 0.9 m) and a rear rotor (Eppler airfoil and D 1.8 m), is shown in Fig. 23.

**Figure 24:**
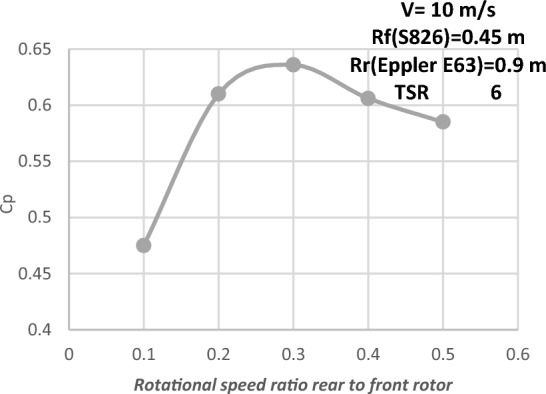
Cp versus rotation speed ratio rear (Eppler E63 D 1.8 m) to front rotor (S826 D 0.9 m) at various N_ratio_ for CWT.

**Figure 25:**
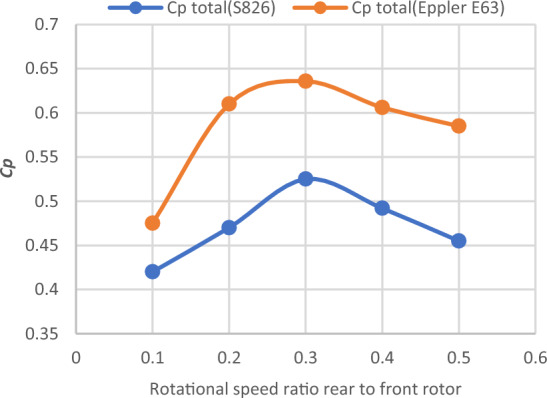
Cp versus rotation speed ratio rear rotor (S826) & (Eppler E63) to front rotor (S826) at various N_ratio_ for CWT.

Investigations are conducted into the DRWT's performance at various N_ratio_ values. The performance coefficient for DRWT employing a diameter ratio of 1:2 and the same type of airfoil is displayed in Table 8 and Fig. 24. The findings indicate that for TSR of 6 and N_ratio_ of 0.3, the case with the best performance (Cp = 0.635) is also achieved. A comparison of the CWT's performance using the S826 and Eppler E63 airfoils is presented in Fig. 25. The findings demonstrate a notable improvement in Cp when compared to the airfoil S826 diameter ratio of 1:2. When the Eppler E63 airfoil is used for the rear rotor, Cp increases by 25.9% at ideal conditions (TSR of 6 and Nratio of 0.3); According to Newman, B^37^ the maximum theoretical CP for the DRWT should be 0.64. The results show how well our design works, with only a 0.65% relative percentage deviation to the maximum limit.

**Table 8:** Performance coefficient for DRWT for diameter ratio of (1:2) with Eppler E63 Airfoil for Rear Rotor at various N_ratio_ values.

TSR	N_ratio_	Cp total
6	0.5	0.585
6	0.4	0.606
6	0.3	0.6358
6	0.2	0.61
6	0.1	0.475

#### Axial separation between the two rotors with Eppler E63 rear rotor (D 1.8)

**Figure 26:**
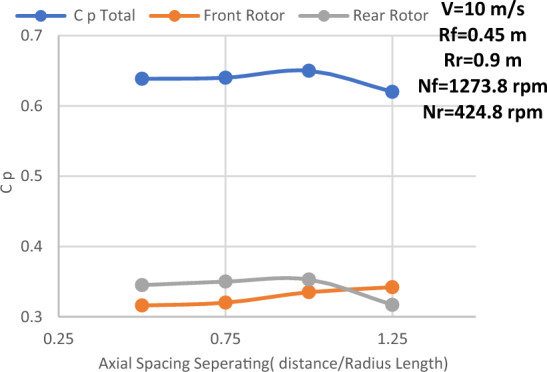
Power coefficient change with axial distance at TSR of 6, N_ratio_ of 0.3, and Eppler E63 airfoil for rear rotor.

**Figure 27:**
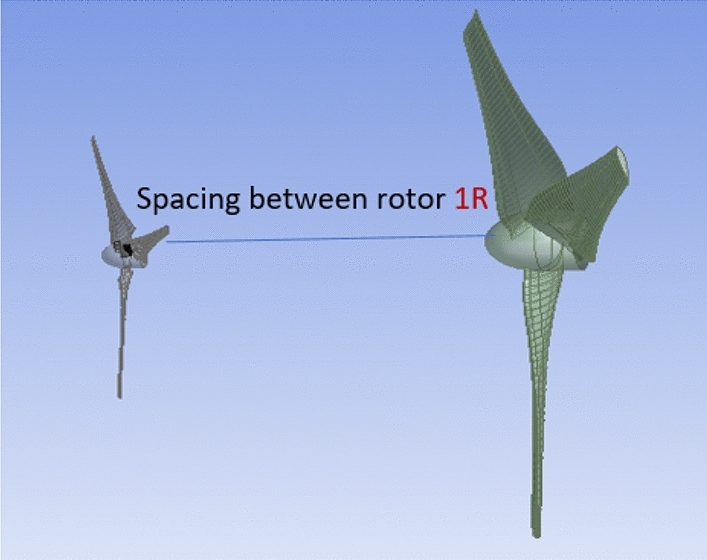
DRWT configuration with front rotor (S826 airfoil and D 0.9 m), rear rotor (Eppler airfoil and D 1.8 m) and an axial distance of 1R_front_.

Figure 26 shows the effectiveness of an Eppler rear rotor with a diameter ratio of 1:2 for different axial separations between the front and rear rotors. At the optimal characteristics for this experiment, the front rotor is S826 airfoil with TSR of 6, the rear rotor is Eppler E63 airfoil with D_front_ of 0.9 m, Drear of 1.8 m, and N_ratio_ of 0.3. The results indicate that the optimal performance happens at an axial distance of 1R_front_ as can be seen in Fig. 27. When compared to the Eppler airfoil used in the preceding section with an axial distance of 0.5Rfront (which has a Cp value of 0.635), the Cp value at this axial distance is 0.0.65, indicating an enhancement of 0.472%. Furthermore, when the axial distance increases, the rear rotor's performance decreases. The rear rotor's reduced performance is a result of its placement outside the wake of the front rotor. As a result, front rotor performance will increase, and interaction between the two rotors will be decreased.

## Conclusion

The effectiveness of SRWT and DRWT designs has been examined in this study. The power output was studied for SRWT and CWT at various TSRs and phase shift angles. According to the SRWT results, the peak Cp value at TSR 6 is the same in both analyses, and the variance is incredibly minimal. When compared to the SRWT, the performance of the CWT was further examined at a N_ratio_ of 1 and a diameter ratio of 1. The CWT's peak performance is reached at a TSR of 4 and the phase shift angle of the rear rotor equals to 5°. The value of Cp at these conditions is 0.513. Moreover, axial distances of 0.25D_front_ or 0.5R of the front rotor yield the optimum overall performance for CWT with Cp of 0.513. However these results demonstrated that the axial separation between the two rotors has no significant effect on CWT at D_ratio_ = 1 and N_ratio_ = 1.

Also, the relationship between rotational speed between the two rotors and the CWT performance was investigated. Moreover, the results revealed that Cp enhanced by 0.575% in comparison to the performance of the diameter ratio of 1:1 when the rear rotor diameter was increased and the diameter ratio changed to 1:2 with the same airfoil type (S826). The results show that when the diameter ratio is altered to 1:2 with an Eppler airfoil for the rear rotor, TSR of 6 and N_ratio_ of 0.3 offered the best performance in this situation. Furthermore, compared to the diameter ratio (1:2) with airfoil S826, the results demonstrate a very large improvement in Cp for both rotors, with a Cp gain of 25.9%. Finally, the findings demonstrate that the performance is at its peak for the influence of axial distance on CWT with Dratio = 1:2 and Eppler airfoil for the rear rotor at an axial distance of 1Rfront and increases by 4.4%. Future research could focus on changing the swirl angle since we believe it's an important parameter to study.

## Data Availability

The corresponding author can provide the datasets created and/or zanalyzed during the current work upon reasonable request.
